# Supplementary material for: Cholesterol mediated ferroptosis suppression reveals essential roles of Coenzyme Q and squalene
Source: Commun Biol. 2023 Nov 1;6:1108. doi: 10.1038/s42003-023-05477-8 (PMC10620397; doi:10.1038/s42003-023-05477-8)

Supplementary Information

Cholesterol mediated ferroptosis suppression reveals essential roles of Coenzyme Q and squalene

Qi Sun[1], Diming Liu[1], Weiwei Cui[2], Huimin Cheng[1], Lixia Huang[1], Ruihao Zhang[1], Junlian Gu[3], Shuo Liu[4], Xiao Zhuang[2], Yi Lu[1*], Bo Chu[2*], Jian Li[1*]

Supplementary Figure 1.
Metabolites of cholesterol biosynthesis pathway exhibit anti-ferroptotic activities.
Supplementary Figure 2.
Characterization of the roles of cholesterol and desmosterol in ferroptosis.
Supplementary Figure 3.
Cholesterol and desmosterol inhibit ferroptosis partly through FSP1-CoQ10 axis.
Supplementary Figure 4.
Both CoQ and squalene are required for cholesterol and desmosterol to suppress ferroptosis.
Supplementary Figure 5.
Cholesterol inhibits ferroptosis in doxorubicin and ischemia-reperfusion induced liver injury in mouse.

Supplementary Methods
Flow cytometry gating strategy.

Uncropped and unedited blot/gel images

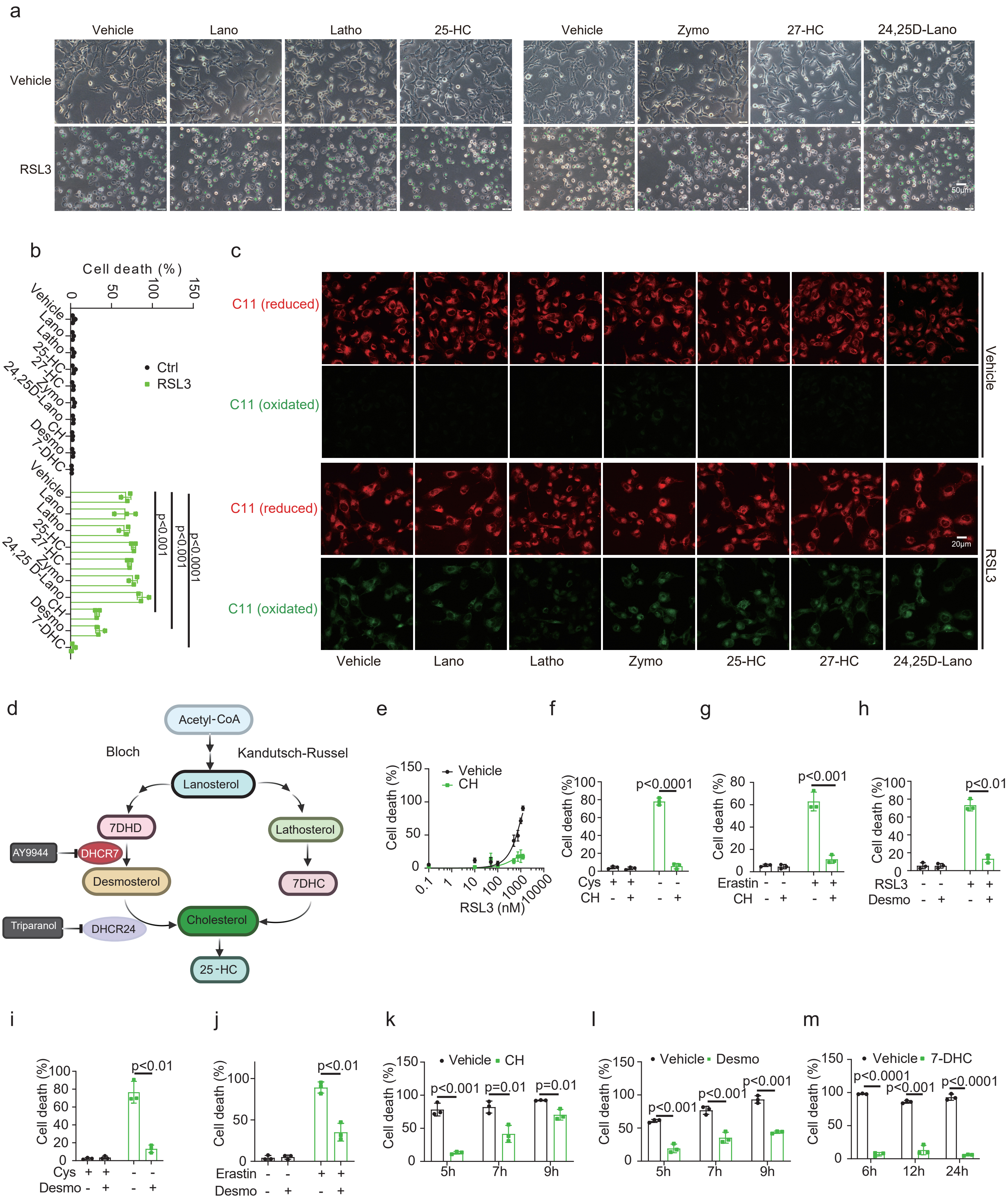

Supplementary Figure 1. Metabolites of cholesterol biosynthesis pathway exhibit anti-ferroptotic activities. **a** Representative phase-contrast images of HT1080 cells in Fig 1**a**. Scale bars, 50μm. **b** Screening and identification of sterol metabolites as ferroptosis inhibitors in 786-O cells. Cells were treated as in Fig.1**a**. **c** Immunofluorescence staining with BODIPY 581/591 C11 to dectect the level of lipid peroxidation in cells treated with Lano (40μM), Latho (40μM), Zymo (40μM), 25-HC (25μM), 27-HC (40μM) and 24, 25D-Lano (40μM).Scale bars, 20μm. **d** Schematic diagram of cholesterol biosynthesis pathway. **e** Dose-dependent toxicity of RSL3 in 786-O cells with or without cholesterol (40μM) treatment. **f** Cell death measurement of 786-O cells cultured in cystine-free medium with cholesterol (40μM) for 20h. **g** Cholesterol suppressed Erastin induced cell death in 786-O cells. See Fig. 1**h** for culture conditions. **h-j** 786-O cells were resistant to RSL3 (**h**), cystine-free medium (**i**), and Erastin (**j**) induced cell death in the presence of desmosterol. Culture conditions were the same with cholesterol treatment. **k-m** Time-dependent cell death induced by RSL3 in HT1080 cells after drug washout: cholesterol (40μM) (**k**), desmosterol (40μM) (**l**), 7-DHC (40μM) (**m**). cells were pretreated with indicated drugs for 3h before washout. RSL3 was added to the cell culture for indicated time to induce ferroptosis. Dead cells were stained with Sytox Green (**b**, **e-m**). Data and error bars are mean ± SD, n = 3 (**b**, **e-m**) independent repeats. Significance in **b** was calculated using a one-way ANOVA with Tukey's post hoc test. Significance in **e-m** was calculated using two-tailed unpaired Student's t-test.

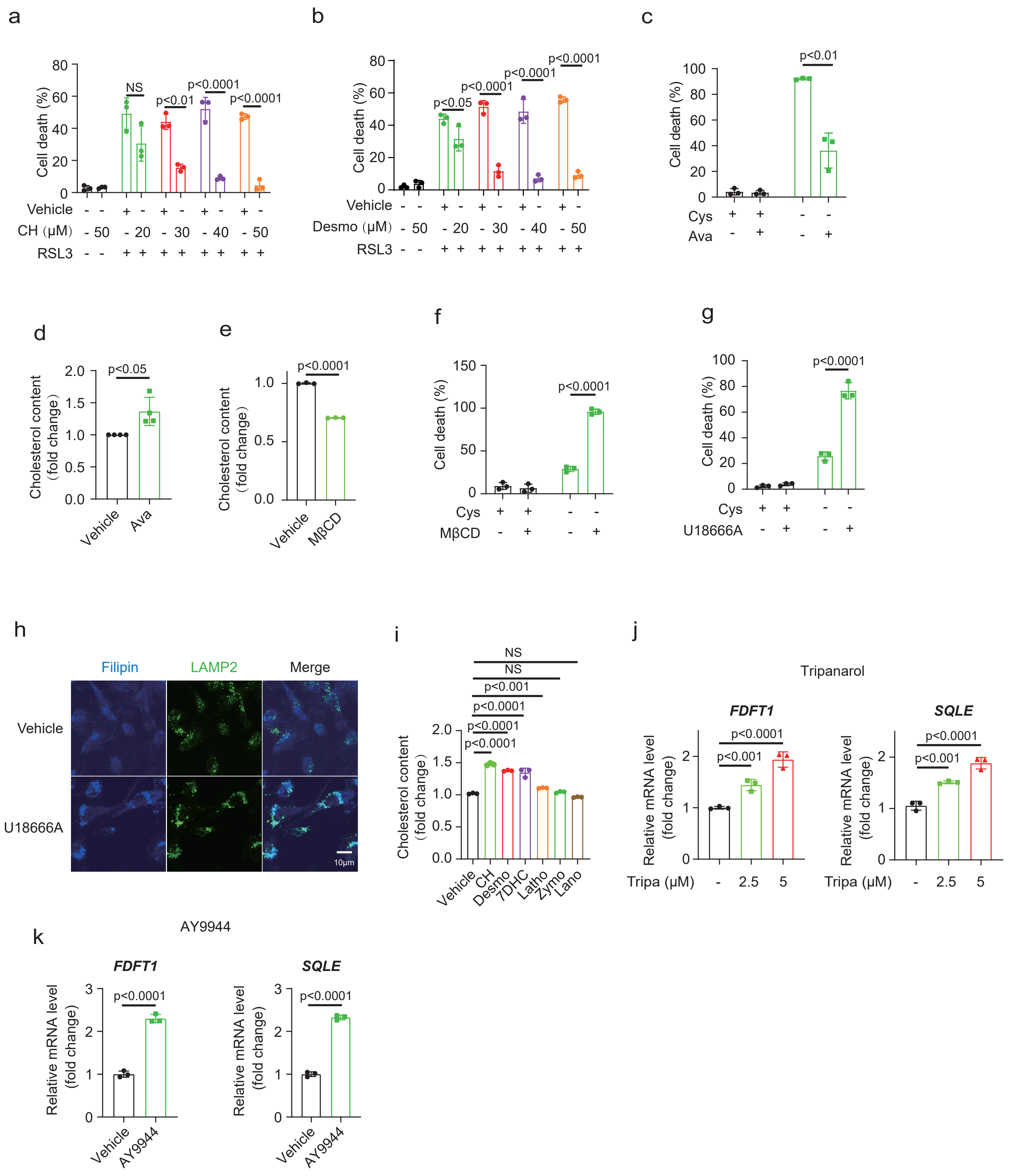

Supplementary Figure 2. Characterization of the roles of cholesterol and desmosterol in ferroptosis. **a** Cell death measurement of HT1080 cells treated with different concentrations of cholesterol and RSL3. Cells were pre-treated with cholesterol with indicated concentrations for 3h before washout, then cells were supplemented with RSL3 (500nM) for 4h. **b** Cell death measurement of HT1080 cells pre-treated with different concentrations of desmosterol and RSL3. See a for culture conditions. **c** Cell death measurement of HT1080 cells pre-treated with avasimible (10μM) for 12h, then co-incubated with cystine-free medium for 24h. **d** Cholesterol content of HT1080 cells treated with avasimible (10μM) for 12h. **e** Cells were cultured in 1% MβCD containing medium for 1h followed by Filipin staining and flow cytometry analysis. **f** Cell death measurement of HT1080 cells treated with or without MβCD and RSL3. Cells were cultured in 1% MβCD containing medium for 30min followed by washout, then cells were treated with cystine-free medium for 17h. **g** Cell death measurement of HT1080 cells co-treated with U18666A (5μM) and cystine-free medium for 17h. **h** Immunofluorescence assay showed accumulation of cholesterol in LAMP2 positive organelles in HT1080 cells. Cells were treated with U18666A (5μM) for 12h. After PFA fixation, cells were stained with 20μg/ml Filipin and LAMP2 antibody, followed by AF488 conjugated secondary antibody (scale bar, 10μm). **i** Filipin staining followed by flow cytometry analysis was used to measure intracellular cholesterol levels. **j** Quantification of mRNA levels of *FDFT1* and *SQLE* in HT1080 cells treated with triparanol (2.5μM and 5μM) for 12h. **k** Quantification of mRNA levels of *FDFT1* and *SQLE* in HT1080 cells treated with AY9944 (5μM) for 12h. Data and error bars are mean ± SD, n = 3 (**a-k**) independent repeats. Significance in **a-g, k** was calculated using two-tailed unpaired Student's t-test. Significance in **i, j** was calculated using a one-way ANOVA with Tukey's post hoc test.

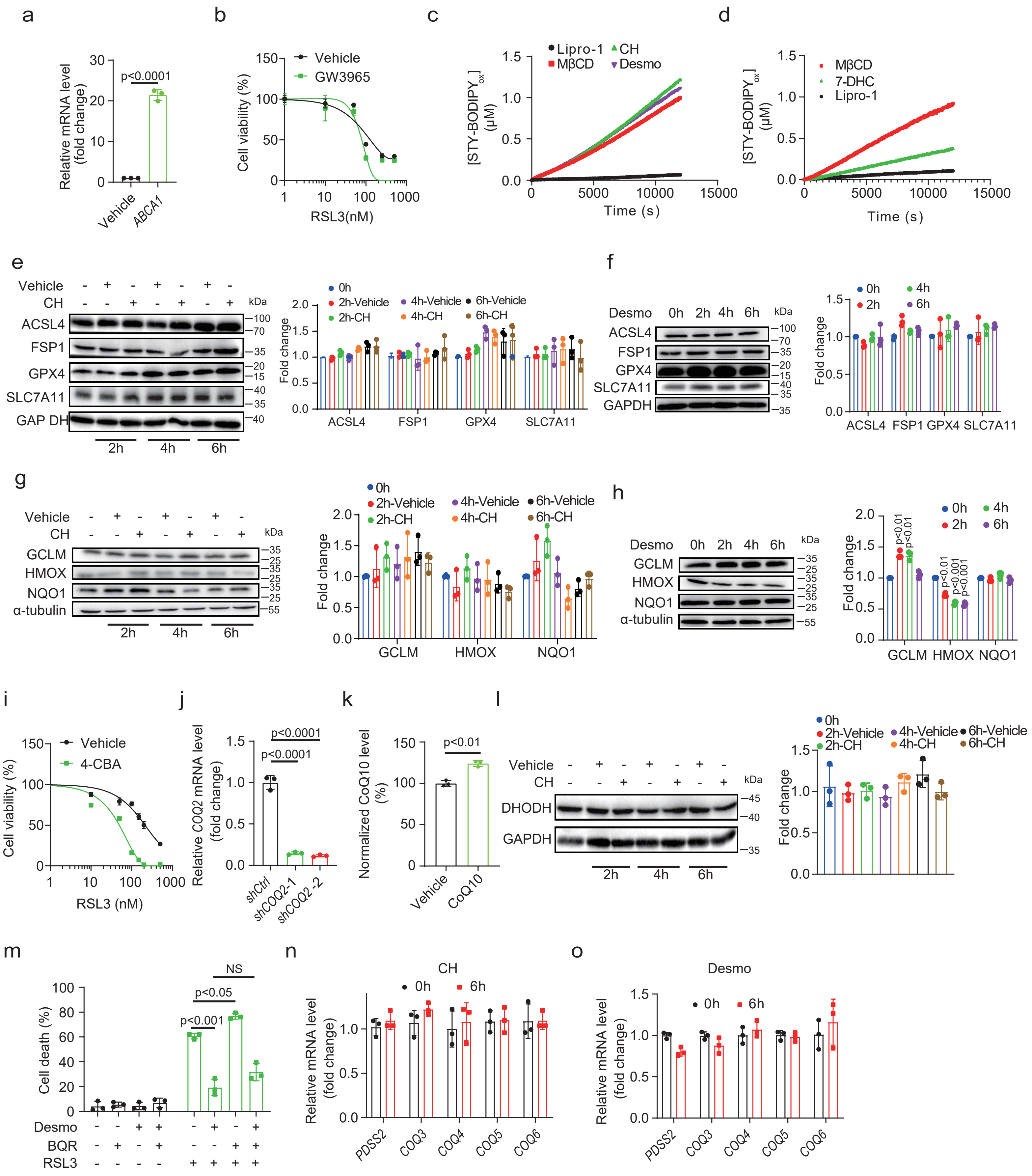

Supplementary Figure 3. Cholesterol and desmosterol inhibit ferroptosis partly through FSP1-CoQ10 axis. a Quantification of mRNA levels of *ABCA1* in HT1080 cells treated with GW3965 (5μM) for 20h. **b** Dose-dependent toxicity of RSL3 in HT1080 cells with or without GW3965 (5μM) treatment. Cell viability was assessed after 20h drug treatment using CCK8 reagent. **c, d** Representative autoxidation of STY-BODIPY (1μM)-embedded liposomes of egg phosphatidylcholine lipids (1mM, ≈100nm particle size). Lipids were suspended in PBS, oxidation was initiated by 0.2 mM DTUN containing lipro-1 (2.5μM) MβCD, cholesterol (40μM), desmosterol (40μM) or 7-DHC (40μM) with the indicated concentrations. **e, g, l** Immunoblot analysis of indicated proteins in HT1080 cells after addition of cholesterol (40μM) for indicated time. MβCD was used as Vehicle control. **f, h** Immunoblot analysis of indicated proteins in HT1080 cells after addition of desmosterol (40μM) for indicated time. **i** Dose-dependent toxicity of RSL3 in HT1080 cells with or without 4-CBA (5mM) treatment. Cell viability was assessed 12h after drug treatment using CCK8 reagent. **j** qPCR analysis of knockdown efficiency of *COQ2* in HT1080 cells. **k** Mass spectrometric analysis of CoQ10 levels of HT1080 cells treated with CoQ10 (250nM) for 12h. **m** Cell death measurement of HT1080 cells pre-treated with RSL3, BQR and desmosterol. See Figure 3g for culture conditions. **n** Quantification of mRNA levels of *PDSS2*, *COQ3*, *COQ4*, *COQ5*, *COQ6* in HT1080 cells treated with cholesterol (40μM) for 6h. **o** Quantification of mRNA levels of *PDSS2*, *COQ3*, *COQ4*, *COQ5*, *COQ6* in HT1080 cells treated with desmosterol (40μM) for 6h. Data and error bars are mean ± SD, n = 3 (**a-o**) independent repeats. Significance in **a, b, i, k, n, o** was calculated using two-tailed unpaired Student's t-test. Significance in **j, m** was calculated using a one-way ANOVA with Tukey's post hoc test.

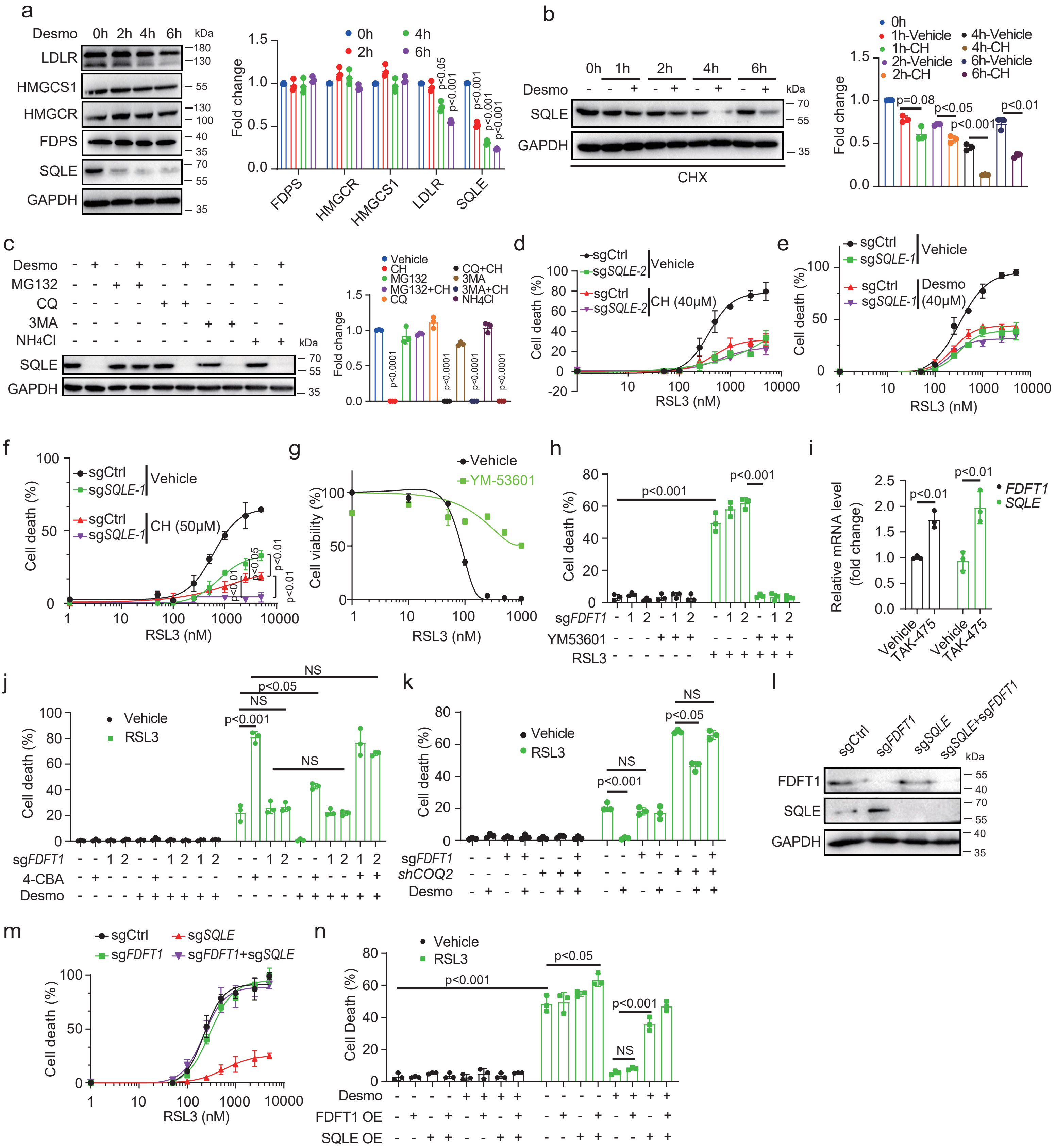

Supplementary Figure 4. Both CoQ and squalene are required for cholesterol and desmosterol to suppress ferroptosis. **a** Immunoblot analysis of indicated proteins in HT1080 cells after addition of desmosterol (40μM) for indicated time. **b** Immunoblot analysis of SQLE protein in HT1080 cells pre-treated with CHX (20ug/ml) for 1h, then co-treated with MβCD/ desmosterol for indicated time. **c** Immunoblot analysis of SQLE protein in HT1080 cells treated with indicated drugs. Inhibitors were pre-treated for 1h and then co-treated with desmosterol (40μM) for 4h. Inhibitors: MG132 (10μM), CQ (10μM), 3MA (5mM), NH4Cl (5mM). **d** Dose-dependent toxicity of RSL3 in HT1080 cells expressing sgRNA-*Ctrl* or sgRNA-*SQLE-2* with or without cholesterol (40μM) treatment. Dead cells were stained with Sytox Green. **e** Dose-dependent toxicity of RSL3 in HT1080 cells expressing sgRNA-*Ctrl* or sgRNA-*SQLE-2* with or without desmosterol (40μM) treatment. **f** Dose-dependent toxicity of RSL3 in HT1080 cells expressing sgRNA-*Ctrl* or sgRNA-*SQLE-2* with or without cholesterol (50μM) treatment. Dead cells were stained with Sytox Green. **g** Dose-dependent toxicity of RSL3 in HT1080 cells with or without YM-53601 (5μM) treatment. Cell viability was assessed after 12h treatment using CCK8 reagent. **h** Cell death measurement of HT1080 cells expressing sgRNA-ctrl or shRNA-*FDFT1* treated with YM-53601 (5μM) for 12h followed by RSL3 (500nM) for 4h. **i** Quantification of mRNA levels of *FDFT1* and *SQLE* in HT1080 cells treated with TAK-475 (5μM) for 12h. **j** Cell death measurement of HT1080 cells transduced with sgRNA-*Ctrl* or sgRNA-*FDFT1*. Cells were pre-treated with 4-CBA (5mM) for 12h, then co-treated with desmosterol (40μM) for 3h, after drug washout, cells were co-treated with 4-CBA (5mM) and RSL3 (500nM) for 3h. Uridine (200μM) was continuously supplemented during the experiment. **k** Cell death measurement of HT1080 cells transduced with sgRNA-*FDFT1* and/or shRNA-*COQ2*. Cells were treated with desmosterol (40μM) for 3h, drugs were washed out, then cells were treated with RSL3 (500nM) for 3h. Uridine (200μM) was continuously supplemented during the experiment. **l** Western blot for FDFT1 and SQLE protein in indicated HT1080 cells. **m** Dose-dependent toxicity of RSL3 in HT1080 cells transduced with sgRNA-ctrl, sgRNA-*FDFT1*, sgRNA-*SQLE* or sgRNA-*FDFT1* + sgRNA-*SQLE*. **n** Cell death measurement of HT1080 cells overexpressing FDFT1, SQLE or FDFT1/SQLE. Cells were treated with desmosterol (40μM) for 3h followed by washout, then cells were treated with RSL3 (500nM) for 4h. Data and error bars are mean ± SD, n = 3 (**a-n**) independent repeats. Significance in **a, c, i** was calculated using two-tailed unpaired Student's t-test. Significance in **b, g, j, k** was calculated using a one-way ANOVA with Tukey's post hoc test. Significance in **f, d, e, h, n** was calculated using a two-way ANOVA with Tukey's post hoc test.

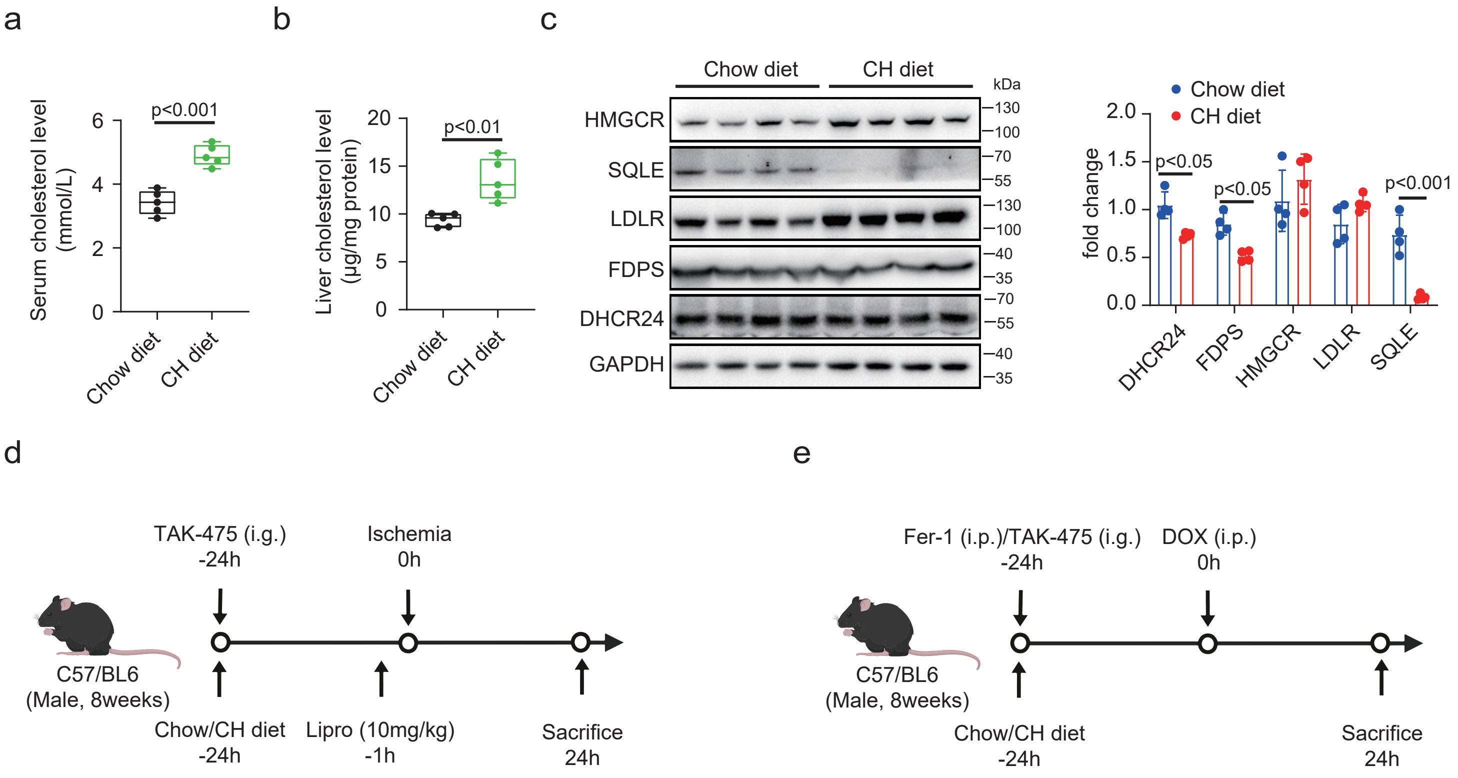

Supplementary Figure 5. Cholesterol inhibits ferroptosis in doxorubicin and ischemia-reperfusion induced liver injury in mouse. **a** cholesterol levels of mice serum **b** and liver tissues (n=5/group). **c** Immunoblot analysis of indicated proteins in mice liver samples after Chow diet or CH diet for 12h (n= 4/group). **d,e** Schematic diagram of mice treatment for IRI or DOX induced liver injury models (n=5/group). Data and error bars are mean ± SD. All P values were calculated using two-tailed unpaired Student's t-test.

Supplementary Methods

Flow cytometry gating strategy.

1. To exclude cell debris, a dot plot graph with forward scatter (FSC)-area (A) vs. the side scatter (SSC)-A was created. A region around the major cell population was selected as P1.
2. The doublet cells were excluded by creating a new dot plot graph from P1 cell population, depicting FSC-height (H) against FSC-width (W). Single cells were selected as P2.
3. The levels of lipid ROS were analyzed through FL1/FL2 channel, and cellular cholesterol levels were analyzed through PB450 in a histogram graph.

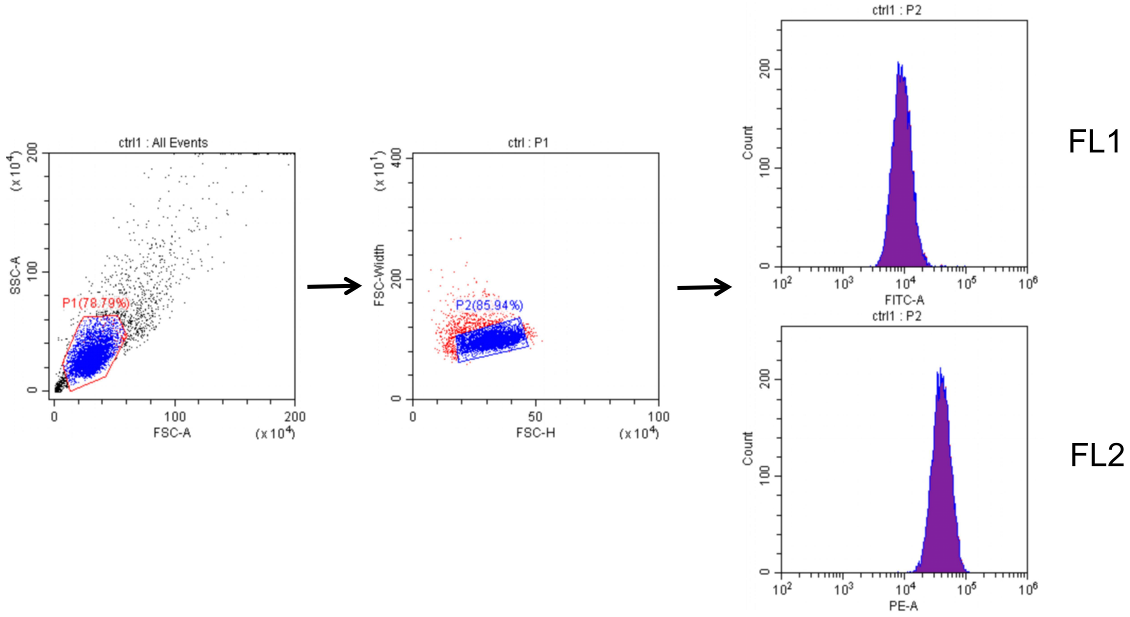

FACS-Based Lipid Peroxidation Assay

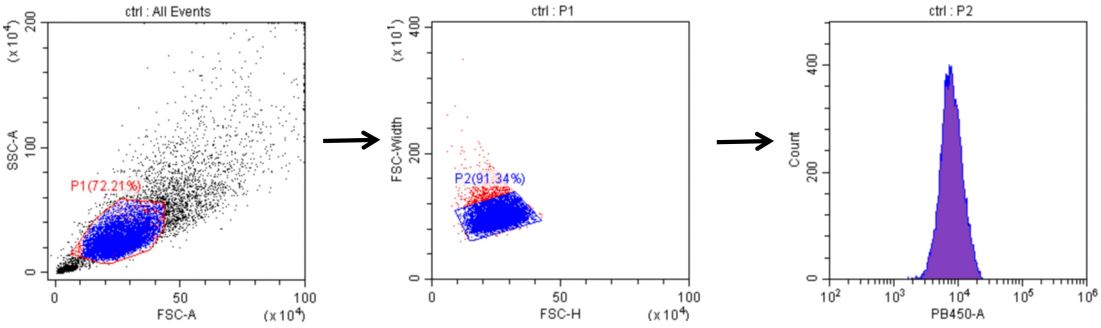

Filipin staining

Figure 1

f

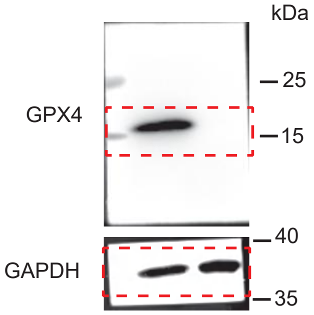

Figure 2

g

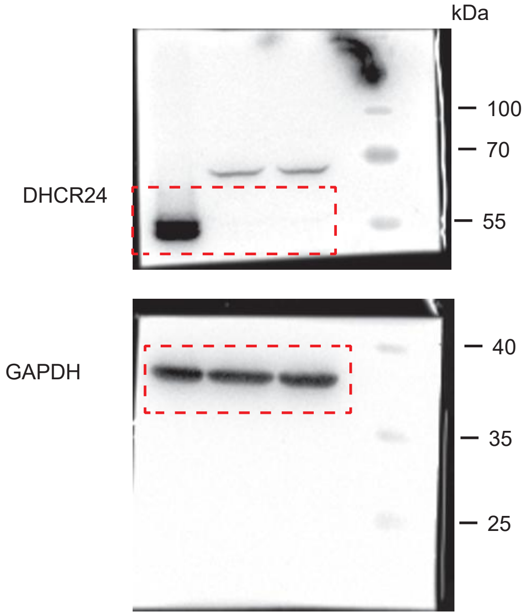
kDa
DHCR24
100
70
55
GAPDH
40
35
25

j

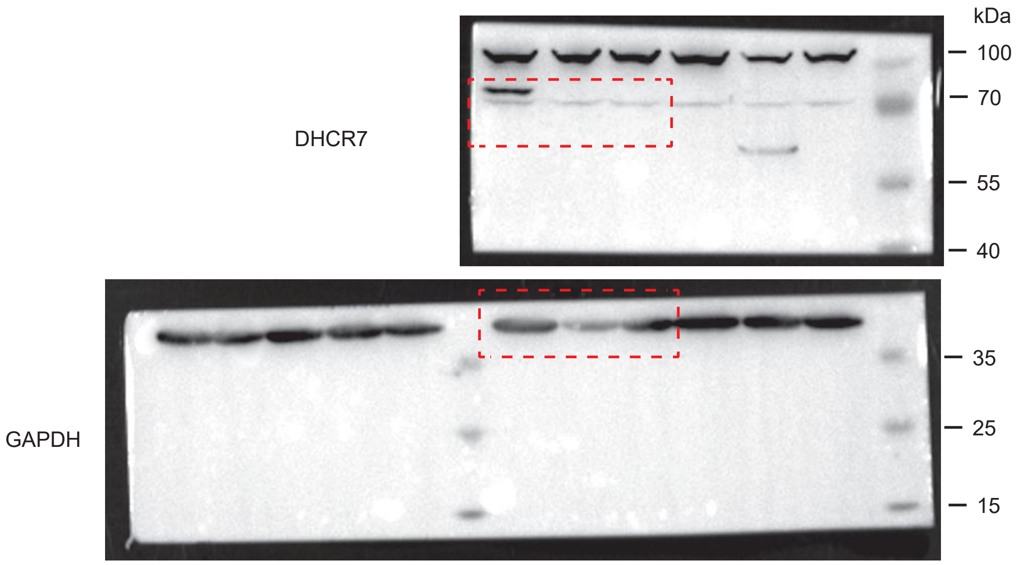
kDa
DHCR7
100
70
55
40
GAPDH
35
25
15

Figure 3

h

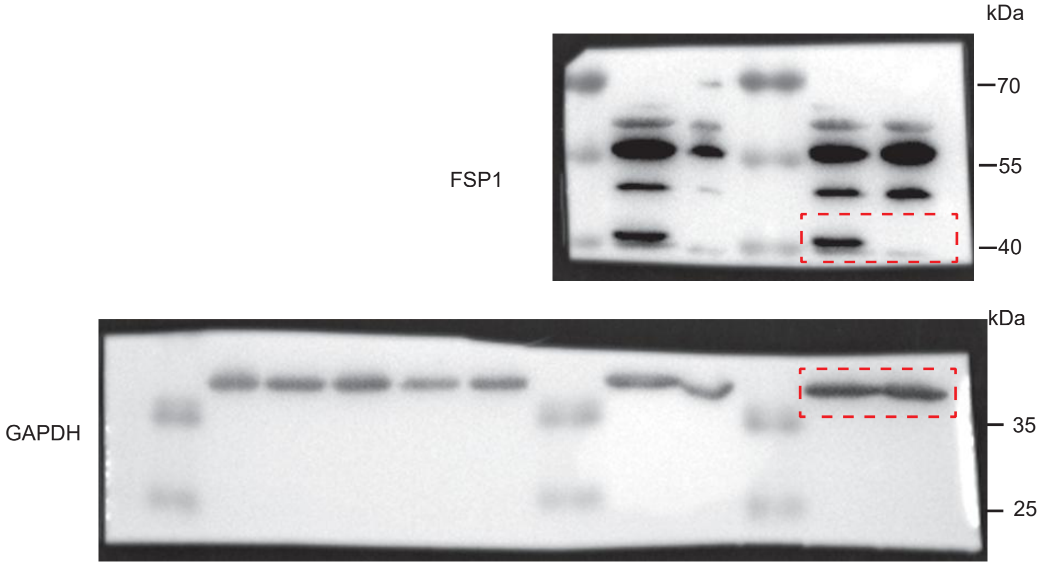

Figure 4

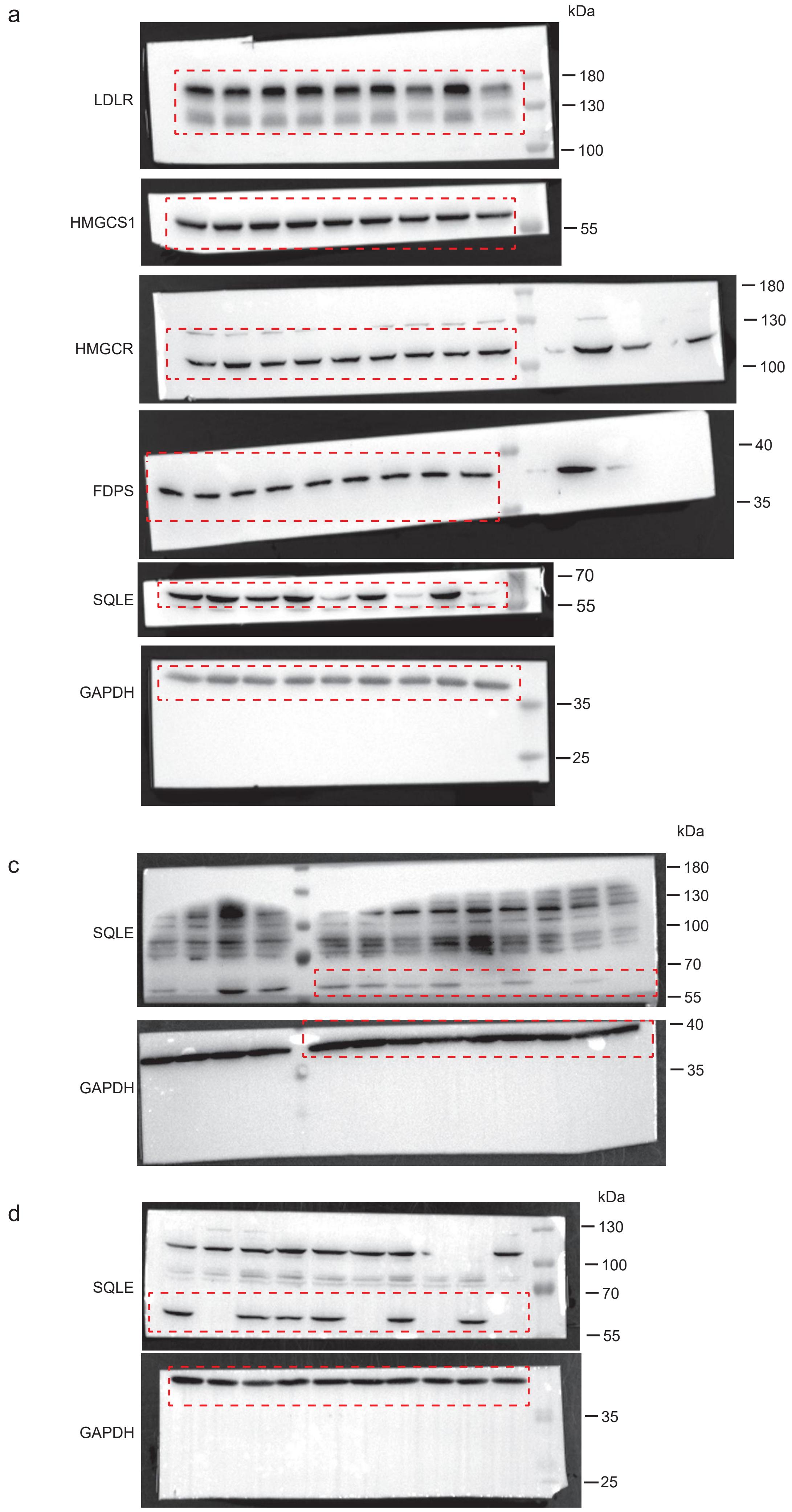
a
kDa
LDLR
180
130
100
HMGCS1
55
HMGCR
180
130
100
FDPS
40
35
SQLE
70
55
GAPDH
35
25
c
kDa
SQLE
180
130
100
70
55
GAPDH
40
35
d
kDa
SQLE
130
100
70
55
GAPDH
35
25

Figure 4

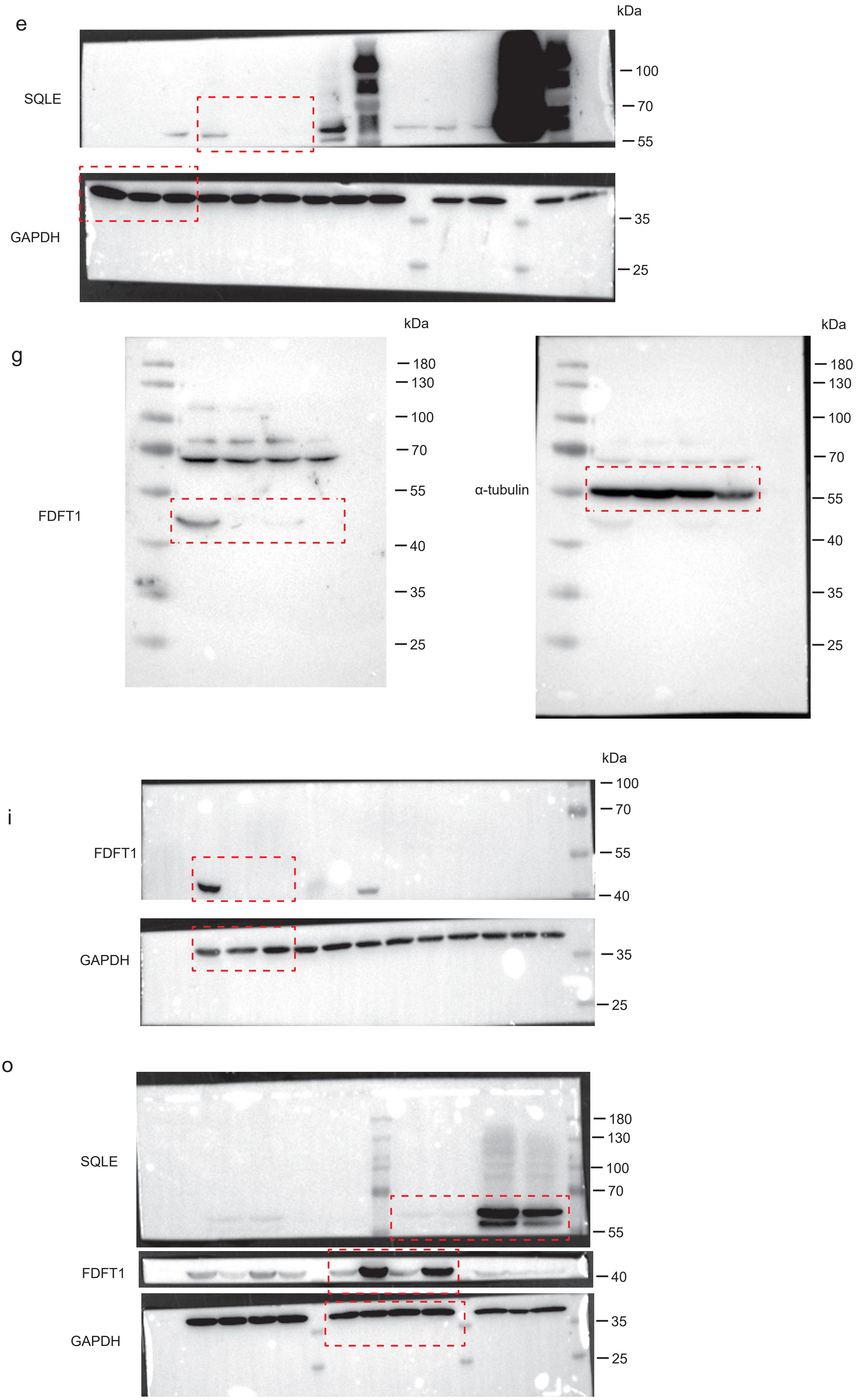
e
kDa
SQLE
100
70
55
GAPDH
35
25
g
FDFT1
kDa
180
130
100
70
55
40
35
25
α-tubulin
kDa
180
130
100
70
55
40
35
25
i
kDa
FDFT1
100
70
55
40
GAPDH
35
25
o
SQLE
180
130
100
70
55
FDFT1
40
GAPDH
35
25

Figure 3-supplementary

e

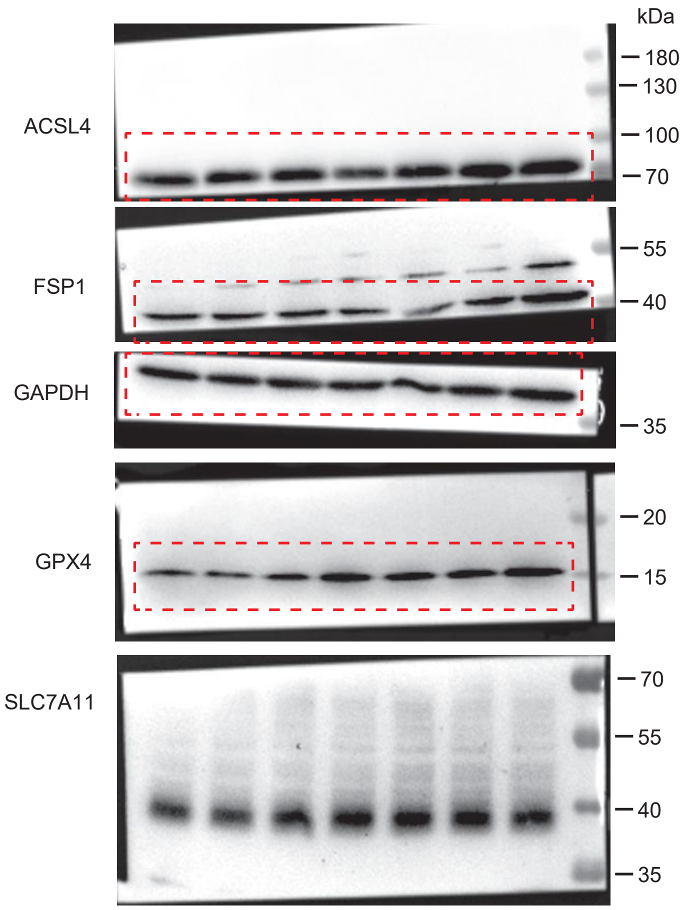

f

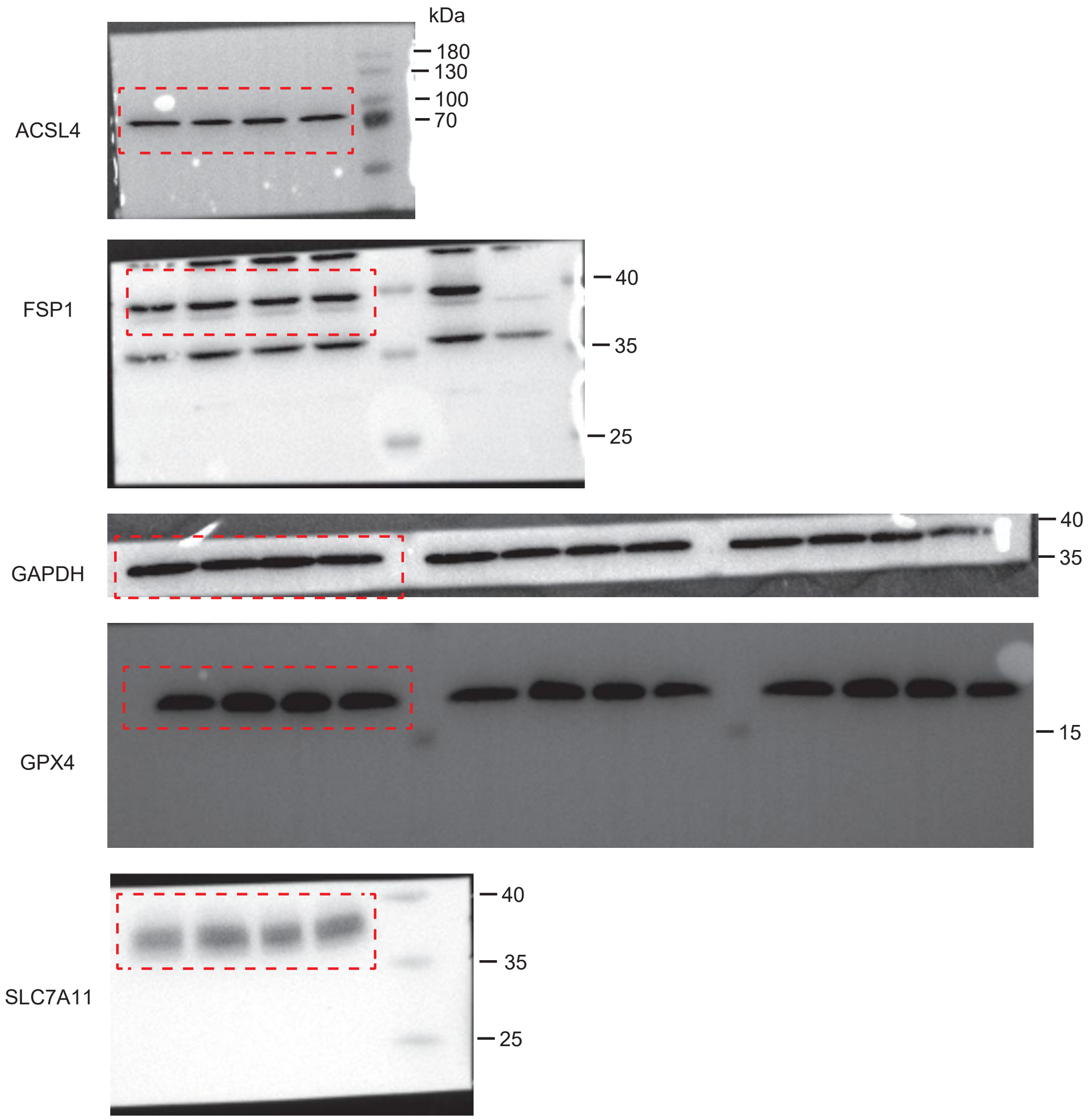

Figure 3-supplementary

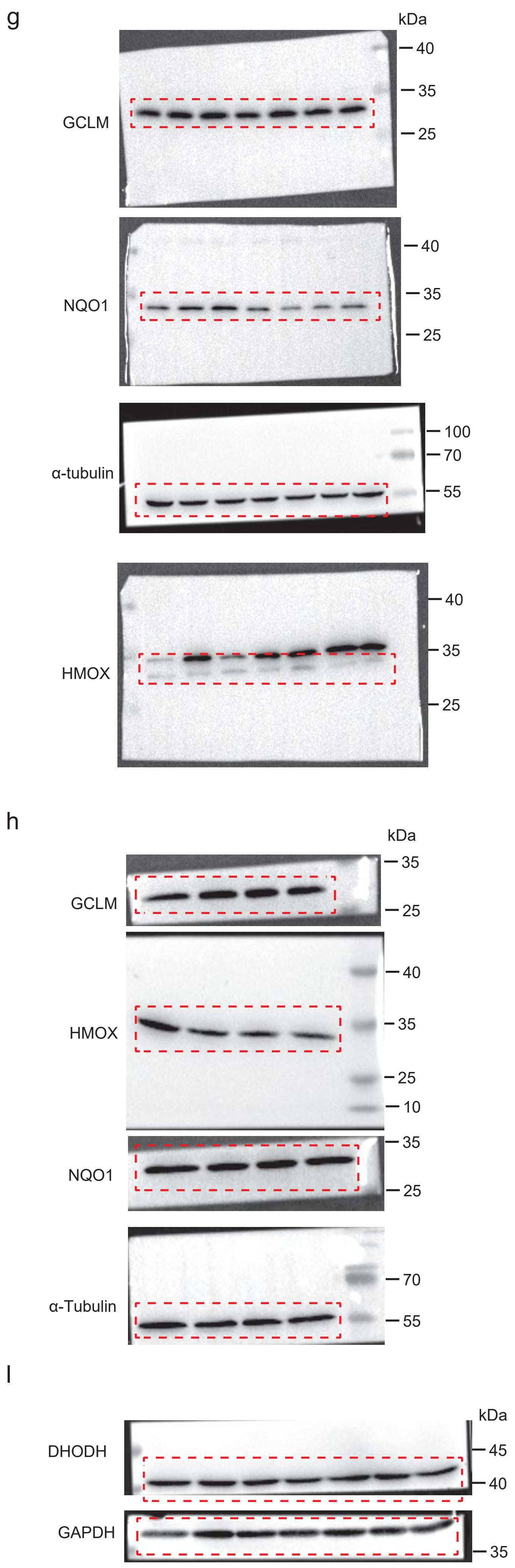

Figure 4-supplementary

a

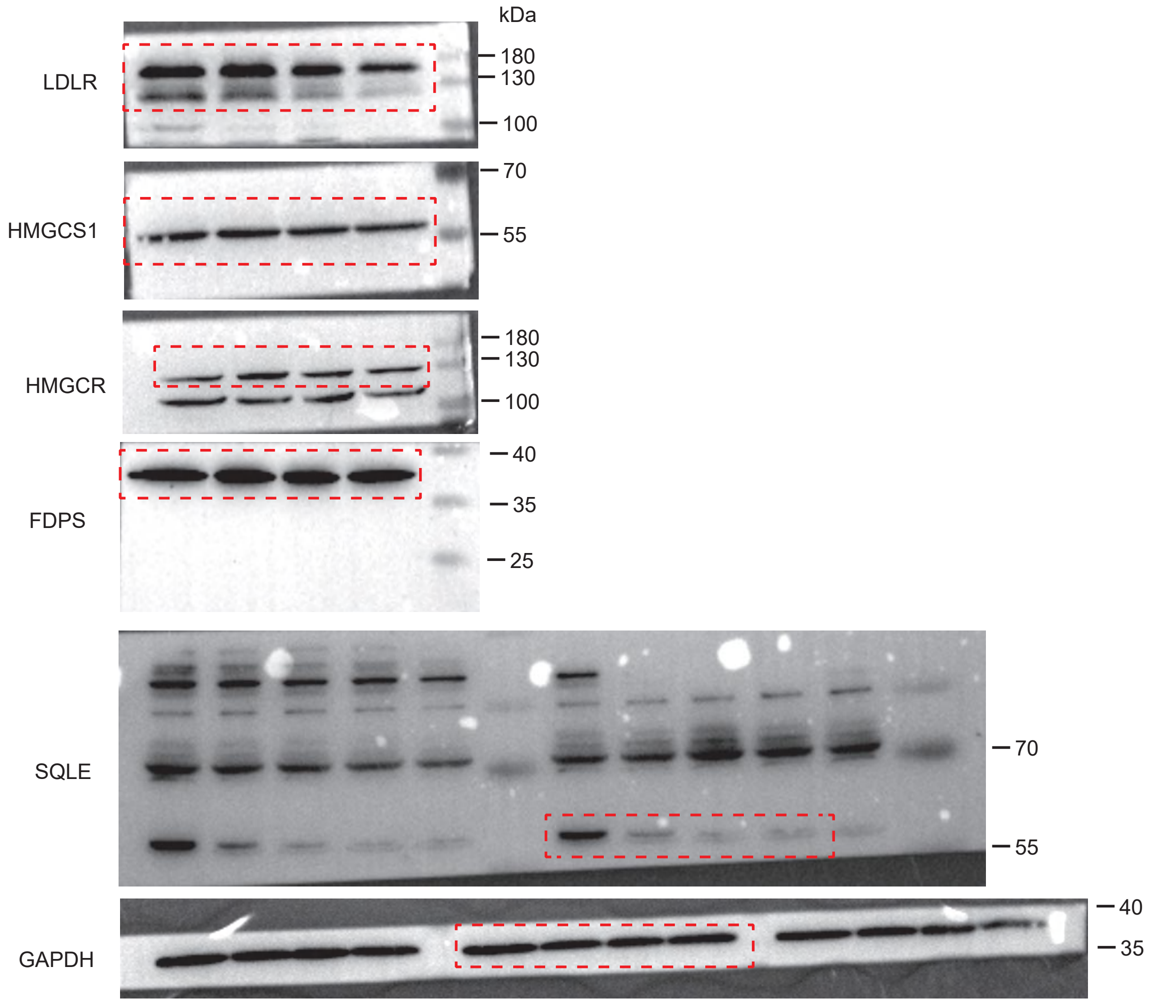

b

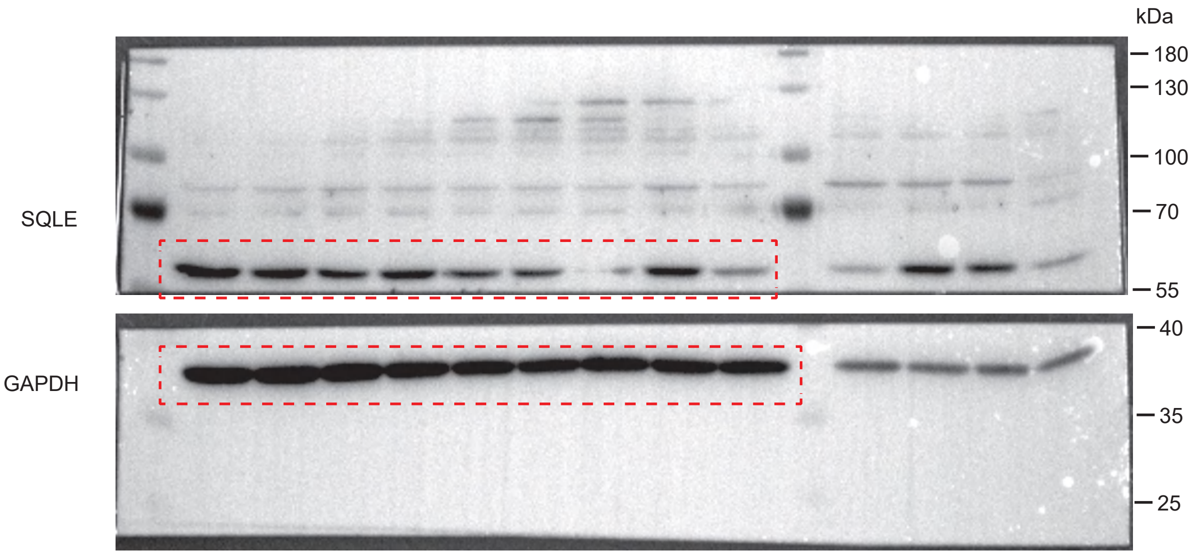

Figure 4-supplementary

c

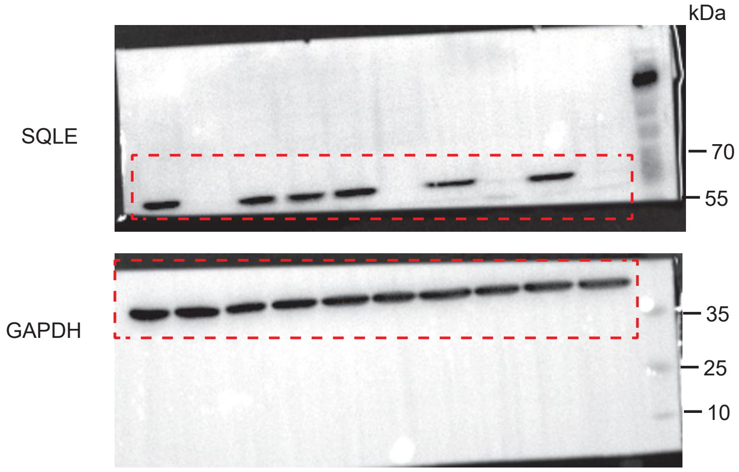

I

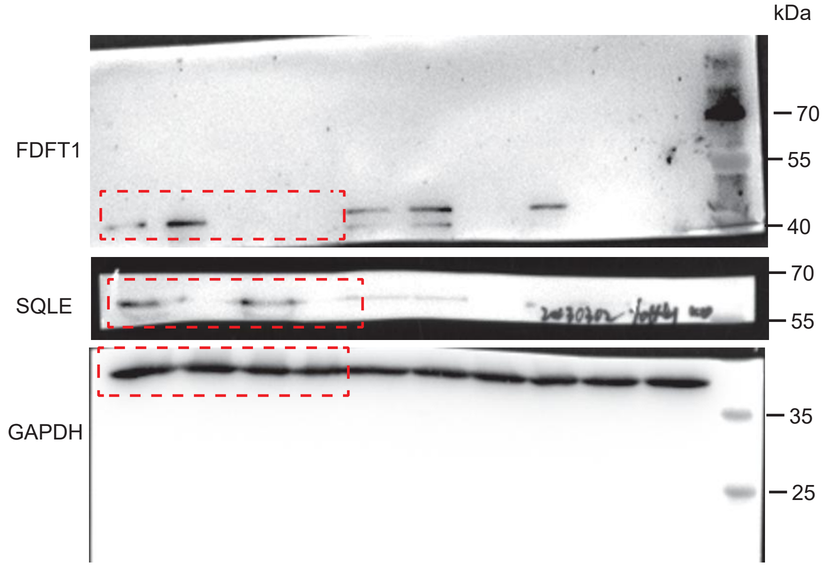

Figure 5-supplementary

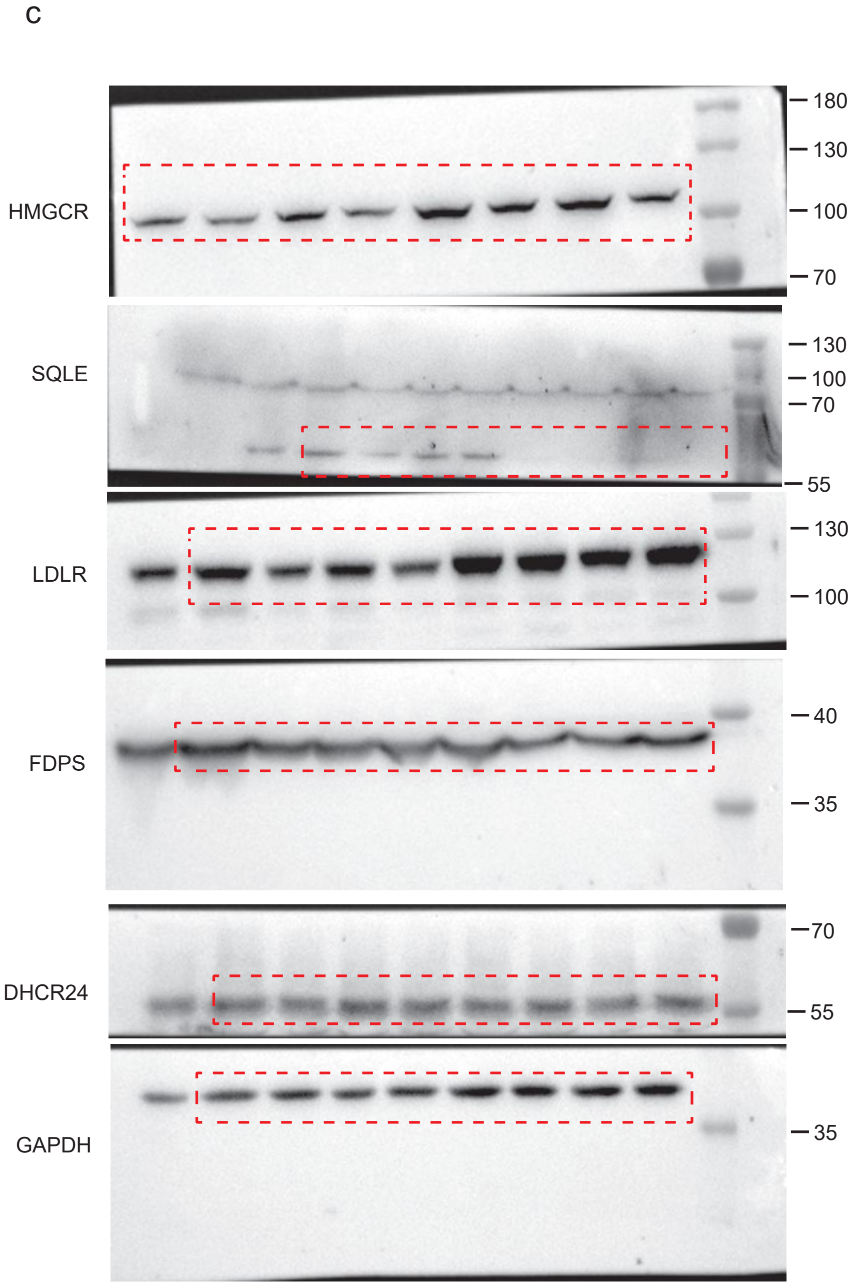

Supplement: Supplementary file 2 — Supplementary Information [file 42003_2023_5477_MOESM2_ESM.pdf]
