# Supplementary material for: Cholesterol mediated ferroptosis suppression reveals essential roles of Coenzyme Q and squalene
Source: Commun Biol. 2023 Nov 1;6:1108. doi: 10.1038/s42003-023-05477-8 (PMC10620397; doi:10.1038/s42003-023-05477-8)
Supplement: Supplementary file 5 — reporting-summary [file 42003_2023_5477_MOESM5_ESM.pdf]

nature portfolio

Corresponding author(s): Jian Li, Bo chu, Yi Lu

Last updated by author(s): Oct 14, 2023

Reporting Summary

Nature Portfolio wishes to improve the reproducibility of the work that we publish. This form provides structure for consistency and transparency in reporting. For further information on Nature Portfolio policies, see our Editorial Policies and the Editorial Policy Checklist.

Statistics

For all statistical analyses, confirm that the following items are present in the figure legend, table legend, main text, or Methods section.

n/a	Confirmed	
☐	☒	The exact sample size (*n*) for each experimental group/condition, given as a discrete number and unit of measurement
☐	☒	A statement on whether measurements were taken from distinct samples or whether the same sample was measured repeatedly
☐	☒	The statistical test(s) used AND whether they are one- or two-sided<br>*Only common tests should be described solely by name; describe more complex techniques in the Methods section.*
☒	☐	A description of all covariates tested
☒	☐	A description of any assumptions or corrections, such as tests of normality and adjustment for multiple comparisons
☐	☒	A full description of the statistical parameters including central tendency (e.g. means) or other basic estimates (e.g. regression coefficient) AND variation (e.g. standard deviation) or associated estimates of uncertainty (e.g. confidence intervals)
☐	☒	For null hypothesis testing, the test statistic (e.g. *F*, *t*, *r*) with confidence intervals, effect sizes, degrees of freedom and *P* value noted<br>*Give P values as exact values whenever suitable.*
☒	☐	For Bayesian analysis, information on the choice of priors and Markov chain Monte Carlo settings
☒	☐	For hierarchical and complex designs, identification of the appropriate level for tests and full reporting of outcomes
☒	☐	Estimates of effect sizes (e.g. Cohen's *d*, Pearson's *r*), indicating how they were calculated

Our web collection on statistics for biologists contains articles on many of the points above.

Software and code

Policy information about availability of computer code

Data collection	Becton Dickinson FACS Calibur machine and Becton cytoflex was used to collect flow cytometry data. Mithras LB940 microplate reader (Berthold Technologies) was used to collect lipid peroxidation level. LC-MS analysis was performed on a Thermo Scientific Dionex Ultimate 3000 UHPLC system hyphenated with a QE-MS equipped with a HESI probe (Thermo Scientific, Bremen, Germany). Sytox Green positive cells were calculated by an inverted fluorescence microscope (Olympus, Japan). Immunohistochemical sections were photographed by upright microscope (Olympus, Japan). QPCR was performed on CFX-Connect machine (BIO-RAD, US). Cholesterol content were calculated by the fluorescence was read by Envision (PerkinElmer, 1905116S).
Data analysis	GraphPad 8 were used for bar graphs output and statistical analysis. FlowJo V10 and CytExpert 2.4 was used for flow cytometry data analysis.

For manuscripts utilizing custom algorithms or software that are central to the research but not yet described in published literature, software must be made available to editors and reviewers. We strongly encourage code deposition in a community repository (e.g. GitHub). See the Nature Portfolio guidelines for submitting code & software for further information.

Data

Policy information about availability of data

All manuscripts must include a data availability statement. This statement should provide the following information, where applicable:

- Accession codes, unique identifiers, or web links for publicly available datasets
- A description of any restrictions on data availability
- For clinical datasets or third party data, please ensure that the statement adheres to our policy

All data that support the conclusions in this manuscript are available from the corresponding author upon reasonable request.

Research involving human participants, their data, or biological material

Policy information about studies with human participants or human data. See also policy information about sex, gender (identity/presentation), and sexual orientation and race, ethnicity and racism.

Reporting on sex and gender	N/A
Reporting on race, ethnicity, or other socially relevant groupings	N/A
Population characteristics	N/A
Recruitment	N/A
Ethics oversight	N/A

Note that full information on the approval of the study protocol must also be provided in the manuscript.

Field-specific reporting

Please select the one below that is the best fit for your research. If you are not sure, read the appropriate sections before making your selection.

☒ Life sciences ☐ Behavioural & social sciences ☐ Ecological, evolutionary & environmental sciences

For a reference copy of the document with all sections, see nature.com/documents/nr-reporting-summary-flat.pdf

Life sciences study design

All studies must disclose on these points even when the disclosure is negative.

Sample size	All the experiments were performed using sample sizes based on standard protocols in the field. In cases where statistics were derived, sample size was n=3 or more independent biological replicates.
Data exclusions	No data were excluded from the analyses.
Replication	Multiple independent repeats were included for related experiments stated in figure legends. Each experiment was performed for at least three times to make sure similar results are reproducible.
Randomization	All mice were randomly allocated into experimental groups.
Blinding	For cell-based experiments, western blotting and FACS, blinding was not possible because the experiments were performed by a single researcher.

Reporting for specific materials, systems and methods

We require information from authors about some types of materials, experimental systems and methods used in many studies. Here, indicate whether each material, system or method listed is relevant to your study. If you are not sure if a list item applies to your research, read the appropriate section before selecting a response.

Materials & experimental systems

n/a	Involved in the study	
☐	☒	Antibodies
☐	☒	Eukaryotic cell lines
☒	☐	Palaeontology and archaeology
☐	☒	Animals and other organisms
☒	☐	Clinical data
☒	☐	Dual use research of concern
☒	☐	Plants

Methods

n/a	Involved in the study	
☒	☐	ChIP-seq
☐	☒	Flow cytometry
☒	☐	MRI-based neuroimaging

Antibodies

Antibodies used

SQLE (12544-1-AP), DHCR24 (10471-1-AP), NQO1 (11451-1-AP), α-Tubulin (11224-1-AP), GAPDH (60004-1-Ig), FSP1 (20886-1-AP), DHODH (14877-1-AP), GCH1 (28501-1-AP) and β-actin (20536-1-AP) were purchased from Proteintech. FDFT1 (A4651), LDLR (A14996), HMGCS1 (A3916), FDPS (A5744) antibodies were purchased from ABclonal. HMGCR (ab174830), GCLM (ab126704), GPX4 (ab125066), ACSL4 (ab155282), 4-HNE (ab46545), MDA (ab243066) antibodies were obtained from abcam. SLC7A11 (#12691) and HO-1 (#70081) antibodies were from CST.
All antibodies were diluted with primary antibody dilution buffer.

Validation

All antibodies used in our study have been validated and detailed information could be obtained the website from manufactures as listed below.
SQLE: https://www.ptgcn.com/products/SQLE-Antibody-12544-1-AP.htm
DHCR24: https://www.ptgcn.com/products/DHCR24-Antibody-10471-1-AP.htm
NQO1: https://www.ptgcn.com/products/NQO1-Antibody-11451-1-AP.htm
α-Tubulin: https://www.ptgcn.com/products/TUBA1B-Antibody-11224-1-AP.htm
GAPDH: https://www.ptgcn.com/products/GAPDH-Antibody-60004-1-Ig.htm
FSP1: https://www.ptgcn.com/products/AIFM2-Antibody-20886-1-AP.htm
DHODH: https://www.ptgcn.com/products/DHODH-Antibody-14877-1-AP.htm
GCH1: https://www.ptgcn.com/products/GCH1-Antibody-28501-1-AP.htm
β-actin: https://www.ptgcn.com/products/ACTB-Antibody-20536-1-AP.htm
FDFT1: https://abclonal.com.cn/catalog/A4651
LDLR:https://abclonal.com.cn/catalog/A14996
HMGCS1: https://abclonal.com.cn/catalog/A3916
FDPS: https://abclonal.com.cn/catalog/A5744
HMGCR: https://www.abcam.cn/products/primary-antibodies/hmgcr-antibody-epr1685n-ab174830.html
GCLM: https://www.abcam.cn/products/primary-antibodies/gclm-antibody-epr6667-ab126704.html
GPX4: https://www.abcam.cn/products/primary-antibodies/glutathione-peroxidase-4-antibody-epncir144-ab125066.html
ACSL4: https://www.abcam.cn/products/primary-antibodies/facl4-antibody-epr8640-ab155282.html
4-HNE: https://www.abcam.cn/products/primary-antibodies/4-hydroxynonenal-antibody-ab46545.html
MDA: https://www.abcam.cn/products/primary-antibodies/malondialdehyde-antibody-11e3-ab243066.html
SLC7A11: https://www.cellsignal.cn/products/primary-antibodies/xct-slc7a11-d2m7a-rabbit-mab/12691?site-search-type=Products&N=4294956287&Ntt=%2312691&fromPage=plp&_requestid=1614852
HO-1: https://www.cellsignal.cn/products/primary-antibodies/ho-1-antibody/70081?site-search-type=Products&N=4294956287&Ntt=%2370081&fromPage=plp&_requestid=1614971

Eukaryotic cell lines

Policy information about cell lines and Sex and Gender in Research

Cell line source(s)

HT1080 and 786-O cell lines were purchased from the Cell Bank of the Chinese Academy of Science (Shanghai, China).

Authentication

Cell line were not authenticated.

Mycoplasma contamination

All cell lines tested negative for mycoplasma contamination.

Commonly misidentified lines
(See ICLAC register)

No ICLAC cell line was used in this study.

Animals and other research organisms

Policy information about studies involving animals; ARRIVE guidelines recommended for reporting animal research, and Sex and Gender in Research

Laboratory animals

8-week old male C57BL/6J mice were purchased from Charles River Laboratories (Beijing, China).

Wild animals

No wild animals involved in this study.

Reporting on sex	Male mice were used in this study.
Field-collected samples	All mice were raised under the pathogen-free mouse facility.
Ethics oversight	All the experiments mice were performed in accordance with a protocol approved by the Policy on the Ethical Use and Care of Animals (School of Basic Medical Sciences, Cheeloo College of Medicine, Shandong University).

Note that full information on the approval of the study protocol must also be provided in the manuscript.

Flow Cytometry

Plots

Confirm that:

☒ The axis labels state the marker and fluorochrome used (e.g. CD4-FITC).

☒ The axis scales are clearly visible. Include numbers along axes only for bottom left plot of group (a 'group' is an analysis of identical markers).

☒ All plots are contour plots with outliers or pseudocolor plots.

☒ A numerical value for number of cells or percentage (with statistics) is provided.

Methodology

Sample preparation	Cells were trypsinized and collected, washed once with PBS. Then each sample was added with 5µM C-11 BODI1PY581/591 dye in PBS and incubated at 37°C for 15 minutes in the dark. Cells were then washed twice with PBS followed by re-suspending in 500µl PBS for flow cytometry analysis.
Instrument	ROS levels were analyzed using a Becton Dickinson FACS Calibur machine through the FL1 and FL2 channel.
Software	The data were analyzed using FlowJo V10 and CytExpert 2.4 software.
Cell population abundance	10， 000 cells were analyzed in each sample.
Gating strategy	Flow cytometry gating strategy.<br>1.To exclude cell debris, a dot plot graph with forward scatter (FSC)-area (A) vs. the side scatter (SSC)-A was created. A region around the major cell population was selected as P1.<br>2.The doublet cells were excluded by creating a new dot plot graph from P1 cell population, depicting FSC-height (H) against FSC-width (W). Single cells were selected as P2.<br>3.The levels of lipid ROS were analyzed through FL1/FL2 channel, and cellular cholesterol levels were analyzed through PB450 in a histogram graph.

☒ Tick this box to confirm that a figure exemplifying the gating strategy is provided in the Supplementary Information.
